# Multi-domain wavelet boundary element method for calculating two-dimensional stress intensity factors

**DOI:** 10.1016/j.heliyon.2024.e29423

**Published:** 2024-04-09

**Authors:** Jiaxing Chen, Dongjie Yuan, Ronggang Yang, Jiawei Xiang

**Affiliations:** a College of Mechanical and Electrical Engineering, Wenzhou University, Wenzhou, 325035, PR China; b Wenzhou Key Laboratory of Dynamics and Intelligent Diagnosis-Maintenance of Advanced Equipment, Wenzhou, 325035, PR China

**Keywords:** Two-dimensional crack problems, Stress intensity factors, Wavelet boundary element method, B-spline wavelet on the interval

## Abstract

In order to improve the accuracy of stress intensity factors (SIFs) calculated by traditional boundary element methods (BEM), the multi-domain wavelet boundary element method (WBEM) is proposed. Firstly, by adjusting the nodes of the B-spline wavelet element on the interval, crack-tip elements are constructed. Since B-spline wavelet on the interval (BSWI) has excellent compact support characteristics and is particularly suitable for describing solution domains with large gradient changes, the constructed crack-tip can reduce the numerical oscillation effect near the crack tip. Secondly, the crack-tip elements are implemented into WBEM. And the combination of WBEM and multi-domain technology can effectively handle interface cracks. Thirdly, the crack problem solving strategy based on multi-domain WBEM can directly evaluate the SIFs of cracks. Finally, several numerical examples involving homogeneous media and bi-material models are given to verify that the proposed method is simple and highly accurate.

## 1 Introduction

In fracture analysis, especially the prediction of crack propagation, it is of particular importance to get an accurate understanding of the stress field near the crack tip. In linear elastic fracture mechanics (LEFM), stress intensity factors (SIFs) are important bases in determining the safety and durability of structures and analyzing how cracks initiate and propagate. However, finding the analytical solution of SIFs is very challenging because of the complexity of structural shapes and boundary conditions, as well as the diversity of crack forms in engineering.

The finite element method (FEM) has gained popularity in the past several decades. In FE analysis, many techniques for crack problems have also been developed, such special crack-tip finite elements [[1], [2], [3]], the extended finite element method (XFEM) [4,5]. The boundary element method (BEM) has dimension reduction characteristics and analytical expression of the field, making it more efficient and accurate in engineering mechanics compared to FEM. Fahmy [[6], [7], [8], [9], [10], [11], [12]] has conducted extensive research on BEM and proposed many techniques for solving complex nonlinear problems, including heat transfer problems, elastic wave propagation problems, structural optimization problems, etc. The boundary-based method naturally describes the singular mechanical behavior near the crack tip, allowing for accurate evaluation of SIF [13]. In the context of two-dimensional gradient elasticity, Karlis et al. [14] analyzed the mixed crack problem of mode I and II using BEM. Nguyen et al. [15] introduced isogeometric analysis into symmetric Galerkin BEM (SGBEM) for solving two-dimensional crack problems. Blandford et al. [16] devised a multi-domain BEM to overcome the problem related with coplanar crack surfaces. Also, the application of multi-domain BEM to handle interface cracks can be found in Refs. [[17], [18], [19]]. Yan [20] constructed a special element with discontinuous crack tip displacement to solve the SIFs. Traction singular quarter-point boundary elements is used on each side of the crack tip, which contain $\sqrt{r}$ displacement and $1/\sqrt{r}$ traction at crack tip [17,16]. These two types of elements are widely used in BEM. However, they cannot accurately obtain oscillatory near-tip solutions in interface cracks. reference [18] verify that the accuracy of SIFs obtained by extrapolation method with BEM under coarse mesh is poor. Some improvement approaches are based on the use of contour integrals and energy release rates. Also, the approximation function of singular field [21] is enriched to characterize the local singular behavior allowing the direct evaluation of the SIFs. As can be seen from the above, the construction of shape functions and node positions have impact on the approximation of singular fields.

In past decades, wavelet numerical methods have become popular to solve engineering problems, especially for problems with singularity and local high gradients, due to their excellent multi-scale, multiresolution and compact support properties [22,23]. The combination of wavelet and finite element analysis for crack problems can be discovered in Refs. [24,25]. Xiang et al. [26] developed interval B-spine wavelet (BSWI) element [[27], [28], [29]]. Compared with traditional wavelet elements, the BSWI element can adapt to complex solution domains and is convenient for element connections and boundary condition processing. Compared to traditional FEM, BSWI FEM can achieve better solution quality in solving crack problems [30]. However, the method retains the low efficiency of mesh generation in FEM. Naturally, the combination of BSWI wavelets and BEM is easy to understand, and its approximation and convergence advantages over traditional BEM can be found in Refs. [[31], [32], [33], [34]]. BSWI has excellent compact support characteristics and is particularly suitable for describing solution domains with large gradient changes. Thus, the WBEM based on BSWI has great appeal in dealing with crack problems, however, there are few reports in this field.

In this paper, the multi-domain WBEM is proposed to solving SIFs in homogeneous and bimaterial crack problems. Firstly, by adjusting the nodes of the BSWI element, crack-tip elements are constructed to reduce the numerical oscillation effect near the crack tip. To the author's knowledge, this type of crack-tip element has not been reported yet. Secondly, the constructed crack-tip elements are implemented into WBEM. And combined with multi-domain method, multi-domain WBEM is proposed to solve interface crack problems. Finally, the numerical solution of the physical field near the tip is substituted into the extrapolation formula to obtain the SIFs. Several crack cases containing homogeneous and bi-material plates are given to validate the proposed method compared with reference solution and BEM method.

## 2 A brief review of BSWI wavelet-based elements for 2D elasticity

To overcome numerical instability of the wavelet approximation on the boundary, Chui and Quak [35] proposed the BSWI on the interval [0,1].

(1) $$\varphi_{m,k}^{j}\left(\xi \right)=\left\{ \begin{array}{cc} \begin{array}{c} \varphi_{m,k}^{l}\left(2^{j-l}\xi \right) \end{array} & \begin{array}{c} k=-m+1,\cdots ,-1\\ \left(0\;\text{boudnary}\;\text{scaling}\;\text{functions}\right) \end{array}\\ \begin{array}{c} \varphi_{m,2^{j}-m-k}^{l}\left(1-2^{j-l}\xi \right) \end{array} & \begin{array}{c} k=2^{j}-m+1,\cdots ,2^{j}-1\\ \left(1\;\text{boudnary}\;\text{scaling}\;\text{functions}\right) \end{array}\\ \begin{array}{c} \varphi_{m,0}^{l}\left(2^{j-l}\xi -2^{-l}k\right) \end{array} & \begin{array}{c} k=0,\cdots ,2^{j}-m\\ \left(\text{inner}\;\text{scaling}\;\text{functions}\right) \end{array} \end{array}\right.$$

Thereafter, the zero scale *m*th-order 1D BSWI scaling functions and wavelet functions are given in Ref. [36]. Then, B-spline scaling functions $\varphi_{m,k}^{j}\left(\xi \right)$ (simply represented as BSWI_mj_) and corresponding wavelet functions $\psi_{m,k}^{j}\left(\xi \right)$, where *j* is scale, can be given by Eq. (1) and Eq. (2).

(2) $$\psi_{m,k}^{j}\left(\xi \right)=\left\{ \begin{array}{cc} \begin{array}{c} \psi_{m,k}^{l}\left(2^{j-l}\xi \right) \end{array} & \begin{array}{c} k=-m+1,\cdots ,-1\\ \left(0\;\text{boundary}\;\text{wavelets}\right) \end{array}\\ \begin{array}{c} \psi_{m,2^{j}-m-k}^{l}\left(1-2^{j-l}\xi \right) \end{array} & \begin{array}{c} k=2^{j}-2m+2,\cdots ,2^{j}-m\\ \left(1\;\text{boundary}\;\text{wavelets}\right) \end{array}\\ \begin{array}{c} \psi_{m,0}^{l}\left(2^{j-l}\xi -2^{-l}k\right) \end{array} & \begin{array}{c} k=0,\cdots ,2^{j}-2m+1\\ \left(\text{inner}\;\text{wavelets}\right) \end{array} \end{array}\right.$$

The 4th order and 3rd scale BSWI_43_ elements have better approximation properties and can achieve high-precision results with a small number of elements, as described in Ref. [31]. Thus, BSWI_43_ element is applied in this paper. For the convenience of describing some basic concepts, BSWI_mj_ is still used here.

**Fig. 1:**
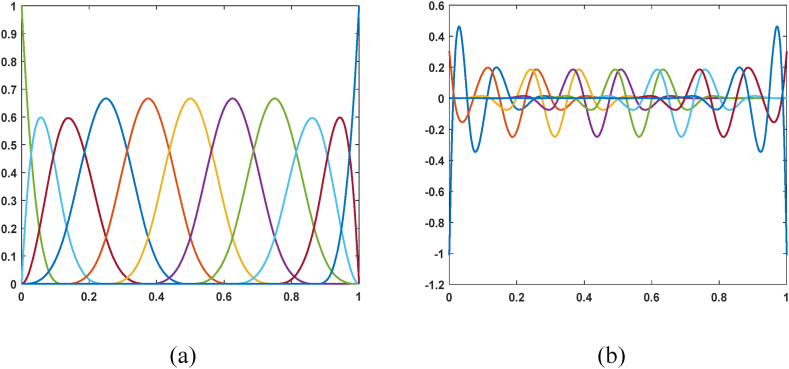
BSWI_43_ on the interval [0,1]: (a) scaling functions, and (b) wave functions.

**Fig. 2:**
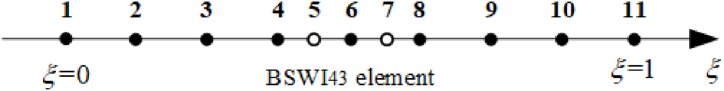
Discrete nodes sequence on BSWI_43_ element.

Fig. 1 shows BSWI_43_ scaling functions and wavelets, and the BSWI_43_ elemental nodes on the interval [0,1] are shown in Fig. 2.

BSWI_mj_ scaling functions (Eq. (1)) is employed as basis functions to construct approximation field. A set of $N=2^{j}+m-1$ BSWI_mj_ functions are written in vector form:

(3) $$\varphi =\left\{\varphi_{m,-m+1}^{j}\left(\xi \right)\;\varphi_{m,-m+2}^{j}\left(\xi \right)\cdots \varphi_{m,2^{j}-1}^{j}\left(\xi \right)\right\}$$

The field variables are approximated by interpolation using BSWI_mj_ functions in 2D linear elastic problems, expressed as

(4) $$\left\{\begin{array}{c} u\\ v \end{array}\right\}=\varphi T^{e}u^{e}$$

where

(5) $$\varphi =\left[\varphi_{m,-m+1}^{j}\left(\xi \right)I\;\varphi_{m,-m+2}^{j}\left(\xi \right)I\cdots \varphi_{m,2^{j}-1}^{j}\left(\xi \right)I\right]$$

(6) $$T^{e}={\left[\varphi^{T}\left(\xi_{1}\right)\;\varphi^{T}\left(\xi_{2}\right)\cdots \varphi^{T}\left(\xi_{N}\right)\right]}^{T}$$

(7) $$u^{e}={\left\{u^{1}\;v^{1}\;u^{2}\;v^{2}\cdots u^{N-1}\;v^{N-1}\;u^{N}\;v^{N}\right\}}^{T}$$

where **I** is the 2 × 2 identity matrix, $T^{e}$ is referred to transformation matrix, u and v are the variable values at nodes along the *x* and *y* coordinate direction, respectively. *N* ($N=2^{j}+m-1$) indicates the number of nodes on BSWI element. The shape function $N^{e}$ can be represented as

(8) $$N^{e}=\varphi T^{e}$$

## 3 Wavelet boundary element method for 2D crack problems

In linear elastic fracture mechanics, the stress intensity factor is related to the field variables in elasticity. For the 2D linear elastic problem, the governing equation and corresponding boundary conditions are given as

(9) $$\left\{ \begin{array}{cc} \sigma_{ij,i}+b_{j}=0 & x_{j}\in \Omega \\ u_{j}={\bar{u}}_{j} & x_{j}\in \Gamma_{1}\\ t_{j}=n_{i}\sigma_{ij}={\bar{t}}_{j} & x_{j}\in \Gamma_{2} \end{array}\right.\;\left(i,j=1,2\right)$$

in which Ω is a solution domain with boundary $\Gamma =\Gamma_{1}\cup \Gamma_{2}$; $x_{j}$ is the coordinate component of a point in the Cartesian coordinate system; $\sigma_{ij,i}$ and $b_{j}$ indicate the component of the body force and the stress tensor, respectively; ${\bar{u}}_{j}$ is the known displacement on the boundary $\Gamma_{1}$; ${\bar{t}}_{j}$ and $n_{i}$ refer to the known traction and the cosine of the outward normal direction on boundary $\Gamma_{2}$, respectively. Using Green formula and Kelvin solution of the elastic problem, without considering the influence of body force, the corresponding boundary integral equation of Eq. (9) is obtained as follows:

(10) $$c_{ji}\left(P\right)u_{i}\left(P\right)=\int_{\Gamma }\left[u_{ji}^{*}\left(P,Q\right)t_{i}\left(Q\right)-t_{ji}^{*}\left(P,Q\right)u_{i}\left(Q\right)\right]d\Gamma \left(Q\right)$$

where P and Q are the source point and the field point and $c_{ji}$ is a coefficient related to the boundary shape at source point, derived as

(11) $$c_{ji}\left(P\right)=\left\{ \begin{array}{cc} \delta_{ji} & P\in \Omega \;\left(\text{inner}\;\text{area}\right)\\ \frac{1}{2}\delta_{ji} & P\in \Gamma \;\left(\text{smooth}\;\text{boundary}\right)\\ \delta_{ji}+\lim_{\varepsilon \to 0}\int_{\Gamma_{\varepsilon }}t_{ji}^{*}d\Gamma & P\in \Gamma \;\left(\text{rough}\;\text{boundary}\right) \end{array}\right.$$

the Kelvin fundamental solution $u_{ji}^{*}$ and $t_{ji}^{*}$ are presented as below:

(12) $$u_{ji}^{*}=\frac{1}{8\pi G\left(1-\nu \right)}\left[\left(3-4\nu \right)\delta_{ji}\;\ln\frac{1}{r}+\frac{\partial r}{\partial x_{j}}\frac{\partial r}{\partial x_{i}}\right]$$

(13) $$t_{ji}^{*}=-\frac{1}{4\pi G\left(1-\nu \right)r}\left\{\frac{\partial r}{\partial n}\left[\left(1-2\nu \right)\delta_{ji}+2\frac{\partial r}{\partial x_{j}}\frac{\partial r}{\partial x_{i}}\right]-\left(1-2\nu \right)\left(\frac{\partial r}{\partial x_{j}}n_{i}-\frac{\partial r}{\partial x_{i}}n_{j}\right)\right\}$$

in which G and ν are the shear modulus and Poisson's ratio of the materials involved, respectively; r=|P−Q| is the Euclidean distance between the source point *P* and field point *Q*; $\delta_{ji}$ is the Kronecker delta function.

The boundary field is approximated by Eq. (4), supposing that *N*_*e*_ BSWI elements are used for boundary Γ and the equation system Eq. (10) is discretized as

(14) $$c_{ji}^{P}u_{i}^{P}+\sum_{e=1}^{N_{e}}\int_{\Gamma_{e}}t_{ji}^{*}\left(P,Q\right)\varphi d\Gamma \left(Q\right)T^{e}u^{e}=\sum_{e=1}^{N_{e}}\int_{\Gamma_{e}}u_{ji}^{*}\left(P,Q\right)\varphi d\Gamma \left(Q\right)T^{e}t^{e}$$

in which $u_{i}^{P}$ is the displacement vector combined with $u_{i}\left(P\right)$; $c_{ji}^{P}$, $t_{ji}^{*}$ and $u_{ji}^{*}$ are the matrix consisting of $c_{ji}\left(P\right)$, $t_{ji}^{*}\left(P,Q\right)$ $u_{ji}^{*}\left(P,Q\right)$, respectively; $u^{e}$ and $t^{e}$ are column vectors composed of displacements and stresses at nodes in the *e*th BSWI_43_ element, respectively. Expand the coefficients within the element according to the degrees of freedom of the nodes, so that Eq. (14) is rewritten as

(15) $$c_{ji}^{P}u_{i}^{P}+\sum_{e=1}^{N_{e}}\left[h_{ji}^{\left(P,e,1\right)},h_{ji}^{\left(P,e,2\right)}\cdots ,h_{ji}^{\left(P,e,N\right)}\right]u^{e}=\sum_{e=1}^{N_{e}}\left[g_{ji}^{\left(P,e,1\right)},g_{ji}^{\left(P,e,2\right)}\cdots ,g_{ji}^{\left(P,e,N\right)}\right]t^{e}$$

where the coefficient block of the *k*-th node in element *e* is derived as

$$h_{ji}^{\left(P,e,k\right)}=\int_{\Gamma_{e}}\varphi_{k}t_{ji}^{*}\left(P,Q\right)d\Gamma \left(Q\right)$$

$$g_{ji}^{\left(P,e,k\right)}=\int_{\Gamma_{e}}\varphi_{k}u_{ji}^{*}\left(P,Q\right)d\Gamma \left(Q\right)$$

Expand the summation term and merge the coefficients of the same DOF, Eq. (15) is written as

(16) $$c_{ji}^{P}u_{i}^{P}+\left[{\hat{h}}_{ji}^{\left(P,1\right)},{\hat{h}}_{ji}^{\left(P,2\right)},\cdots ,{\hat{h}}_{ji}^{\left(P,NP\right)}\right]\left\{U\right\}=\left[g_{ji}^{\left(P,1\right)},g_{ji}^{\left(P,2\right)}，\cdots ,g_{ji}^{\left(P,NP\right)}\right]\left\{T\right\}$$

where **U** and **T** are column vectors combined by displacements and stresses at all nodes, respectively. Furthermore, Eq. (16) can be represented as

(17) $${\left[h\right]}_{2\times 2NP}{\left\{U\right\}}_{2NP\times 1}={\left[g\right]}_{2\times 2NP}{\left\{T\right\}}_{2NP\times 1}$$

where the coefficient in [h] can be obtained as

$$h_{ji}^{PQ}=\left\{ \begin{array}{cc} {\hat{h}}_{ji}^{PQ} & P\neq Q\;\\ {\hat{h}}_{ji}^{PQ}+c_{ji}^{P} & P=Q \end{array}\right.$$

Taking all boundary nodes as the source point and substituting them into Eq. (17), and the linear algebraic equation is obtained as

(18) $${\left[H\right]}_{2NP\times 2NP}{\left\{U\right\}}_{2NP\times 1}={\left[G\right]}_{2NP\times 2NP}{\left\{T\right\}}_{2NP\times 1}$$

According to the load on the model and the known parameters of the node, the following equation can be obtained:

(19) $${\left[A\right]}_{2NP\times 2NP}{\left\{X\right\}}_{2NP\times 1}={\left\{F\right\}}_{2NP\times 1}$$

where Χ is a column vector including unknown values of nodes on the boundary, F is the known vector on the right-hand side. The displacement components and stress components of all nodes on the boundary can be obtained by solving Eq. (19).

## 4 The treatment for the 1/randIn(1/r) singular integrals

The singular integrals in this paper mainly involve strongly singular of 1/r and weakly singular of In(1/r). There are many effective methods [[37], [38], [39]] for handling singular integrals. The rigid body displacement method is very convenient for indirectly handling strong singular integrals. Then the strongly singular integral term in Eq. (18) can be calculated as

(20) $$h_{ji}^{PP}=-\sum_{\begin{array}{c} P=1\\ P\neq Q \end{array}}^{NP}h_{ji}^{PQ}$$

(21) $$h_{ji}^{PP}=I-\sum_{\begin{array}{c} P=1\\ P\neq Q \end{array}}^{NP}h_{ji}^{PQ}$$

where Eq. (20) and Eq. (21) are used to handle finite and infinite fields, respectively. Weakly singular integrals are processed by logarithmic numerical integrals, for which we need to transform the integration domain to [0,1]. As BSWI is a piecewise function, we take a region $\xi^{a}=\left[\xi_{1}^{a},\xi_{2}^{a}\right]$ as a demonstration of integration. The corresponding integration region of the physical domain is denoted as $\Gamma^{a}=\left[x_{1}^{a},x_{2}^{a}\right]\times \left[y_{1}^{a},y_{2}^{a}\right]$. Then, a weakly singular integral form in physical domain is given as

(22) $$\int_{\Gamma^{a}}f\left(x,y\right)\text{In}\left(\frac{1}{r}\right)d\Gamma$$

Similar to linear BEM element, a linear local transformation is used for geometric approximations. Next, we will consider three scenarios in the parameter domain where the point source is on the upper bound, lower bound, and inner of the integral domain, denoted as A, B, and C. In case A, the linear transformation is given as

(23) $$\left\{ \begin{array}{c} x=x_{1}^{a}+\left(x_{2}^{a}-x_{1}^{a}\right)\xi \\ y=y_{1}^{a}+\left(y_{2}^{a}-y_{1}^{a}\right)\xi \end{array}\right.$$

Then Eq. (22) can be deduced as

(24) $$\int_{\Gamma^{a}}\;f\left(x,y\right)\text{In}\left(\frac{1}{r}\right)d\Gamma =\int_{0}^{1}\;f\left(x\left(\xi \right),y\left(\xi \right)\right)\text{In}\left(\frac{1}{C\xi }\right)Jd=\int_{0}^{1}f\left(\xi \right)\text{In}\left(\frac{1}{C}\right)Jd\xi +\int_{0}^{1}f\left(\xi \right)\text{In}\left(\frac{1}{\xi }\right)Jd\xi \xi$$

where $J=C=\sqrt{{\left(x_{2}^{a}-x_{1}^{a}\right)}^{2}+{\left(y_{2}^{a}-y_{1}^{a}\right)}^{2}}$ and the first and second terms on the right side of Eq. (24) can be calculated by Gaussian and logarithmic numerical integration, respectively. In case B, the linear transformation is given as

(25) $$\left\{ \begin{array}{c} x=x_{2}^{a}+\left(x_{1}^{a}-x_{2}^{a}\right)\xi \\ y=y_{2}^{a}+\left(y_{1}^{a}-y_{2}^{a}\right)\xi \end{array}\right.$$

Then Eq. (22) can be derived as follow:

(26) $$\int_{\Gamma^{a}}\;f\left(x,y\right)\text{In}\left(\frac{1}{r}\right)d\Gamma =\int_{0}^{1}\;f\left(x\left(\xi \right),y\left(\xi \right)\right)\text{In}\left(\frac{1}{C\xi }\right)Jd\xi =\int_{0}^{1}f\left(\xi \right)\text{In}\left(\frac{1}{C}\right)Jd\xi +\int_{0}^{1}f\left(\xi \right)\text{In}\left(\frac{1}{\xi }\right)Jd\xi$$

Subsequently, Eq. (26) can be processed in the same way as Eq. (24). For case 3, the integration domain is divided into two parts $\xi^{a_{1}}=\left[\xi_{1}^{a},\xi_{P}^{a}\right]$ and $\xi^{a_{2}}=\left[\xi_{P}^{a},\xi_{2}^{a}\right]$ based on the point source, so that the point *P* is at the lower and upper bound of the corresponding integration domain. Then the integrals can be processed according to Eqs. (24), (26), respectively.

## 5 Multi-domain WBEM for crack problem

### 5.1 Multi-domain WBEM

An elastic body with cracks is divided into two subdomains, in which the boundary integral equation for each subdomain $\Omega_{l}$ is given by Eq. (10).

(27) $$c_{ji}^{\left(\Omega_{l}\right)}\left(P\right)u_{i}^{\left(\Omega_{l}\right)}\left(P\right)+\int_{\Gamma^{\left(\Omega_{l}\right)}}{u_{ji}^{*}}^{\left(\Omega_{l}\right)}\left(P,Q\right)t_{i}^{\left(\Omega_{l}\right)}\left(Q\right)d\Gamma^{\left(\Omega_{l}\right)}\left(Q\right)=\int_{\Gamma^{\left(\Omega_{l}\right)}}{t_{ji}^{*}}^{\left(\Omega_{l}\right)}\left(P,Q\right)u_{i}^{\left(\Omega_{l}\right)}\left(Q\right)d\Gamma^{\left(\Omega_{l}\right)}\left(Q\right)\;l=1,2$$

Then the boundary conditions on the shared interface *I* of the subdomain are given as

(28) $$u_{I}^{\Omega_{1}}=u_{I}^{\Omega_{2}}\;t_{I}^{\Omega_{1}}=-t_{I}^{\Omega_{2}}$$

**Fig. 3:**
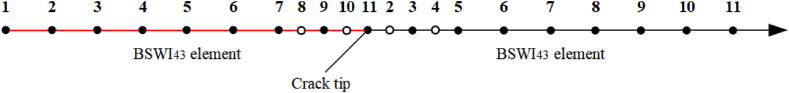
WBEM nodes for field variables near crack-tip in parameter domain.

Due to the singularity of stress near the crack tip, it is necessary to treat the crack tip elements. Here, BSWI_43_ element near the crack tip is simply adjusted, and the corresponding nodes in parameter domain are shown in Fig. 3. There are more nodes near the crack tip compared to other regions of the element, which is inspired by the idea of local refinement to handle singular regions.

The crack-tip BSWI_43_ elements and other BSWI_43_ elements (seen in Fig. 2) are used to discretize the boundary in Eq. (27). Applying the processing in the third and fourth sections, the solution matrix equation for each subdomain is obtained as

(29) $$\left[H^{1},H_{I}^{1}\right]\left(\begin{array}{c} \left\{u^{1}\right\}\\ \left\{u_{I}^{1}\right\} \end{array}\right)=\left[G^{1},G_{I}^{1}\right]\left(\begin{array}{c} \left\{t^{1}\right\}\\ \left\{t_{I}^{1}\right\} \end{array}\right)$$

(30) $$\left[H^{2},H_{I}^{2}\right]\left(\begin{array}{c} \left\{u^{2}\right\}\\ \left\{u_{I}^{2}\right\} \end{array}\right)=\left[G^{2},G_{I}^{2}\right]\left(\begin{array}{c} \left\{t^{2}\right\}\\ \left\{t_{I}^{2}\right\} \end{array}\right)$$

Impose boundary conditions of Eq. (28), the matrix equations of all subdomains are coupled together as

(31) $$\left[\begin{array}{ccc} H^{1} & H_{I}^{1} & 0\\ 0 & H_{I}^{2} & H^{2} \end{array}\right]\left(\begin{array}{c} \left\{u^{1}\right\}\\ \left\{u_{I}\right\}\\ \left\{u^{2}\right\} \end{array}\right)=\left[\begin{array}{ccc} G^{1} & G_{I}^{1} & 0\\ 0 & -G_{I}^{2} & G^{2} \end{array}\right]\left(\begin{array}{c} \left\{t^{1}\right\}\\ \left\{t_{I}\right\}\\ \left\{t^{2}\right\} \end{array}\right)$$

where $u_{I}=u_{I}^{\Omega_{1}}=u_{I}^{\Omega_{2}}$ and $t_{I}=t_{I}^{\Omega_{1}}=-t_{I}^{\Omega_{2}}$. By solving Eq. (31), the unknown displacements and stresses at all nodes are determined. For other possible cracks in the elastic body, more subdomains are divided. Then, in the same way, the matrix equations of all subdomains are added to the system and solved.

### 5.2 Calculation of the SIF

There are many methods for evaluating stress intensity factors [17,18,40,41]. The extrapolation method can avoid oscillation singularity and is applied in this paper. The mode I SIF $K_{1}$ is given [40] as

(32) $$K_{1}=\frac{2G}{\kappa +1}\sqrt{\frac{2\pi }{r}}v$$

in which *v* is the displacement solution at crack tip; *r* represent the distance between the crack tip and the node under investigation; *G* and κ are the shear modulus the elastic parameter (κ=(3−μ)/(1+μ) for the plane stress and κ=3−4μ for the plane strain).

In the bimaterial interface crack problem, the complex formula for calculating the SIF based on stress field is obtained as

(33) $$K=K_{1}+iK_{2}=\lim_{r\to 0}\left\{\sqrt{2\pi r}\left(\sigma_{y}+i\tau_{xy}\right)/r^{i\varepsilon }\right\}$$

in which $\sigma_{y}$ and $\tau_{xy}$ are stress solution near crack tip; $i=\sqrt{-1}$ and $K_{2}$ is the mode II SIF. ε is oscillation index related to the material, expressed as

(34) $$\varepsilon =\frac{1}{2\pi }\ln\frac{1-\beta }{1+\beta }$$

where

$$\beta =\frac{G_{1}\left(\kappa_{2}-1\right)-G_{2}\left(\kappa_{1}-1\right)}{G_{1}\left(\kappa_{2}+1\right)+G_{2}\left(\kappa_{1}+1\right)}$$

in which $G_{1}$ and $G_{2}$ are the shear modulus of material 1 and material 2, respectively; $\kappa_{1}$ and $\kappa_{2}$ is the elastic parameter of material 1 and material 2, respectively.

According to Euler's formula, $r^{i\varepsilon }$ in Eq. (33) is deduced as

(35) $$r^{i\varepsilon }=\cos\left(\varepsilon \;\ln\;r\right)+i\;\sin\left(\varepsilon \;\ln\;r\right)$$

Combined with Eq. (35) and Eq. (33), $K_{1}$ and $K_{2}$ in Eq. (33) are decoupled and independently represented as

(36) $$\begin{array}{c} K_{1}=\lim_{r\to 0}\left\{\sqrt{2\pi r}\left[\sigma_{y}\;\cos\left(\varepsilon \;\ln\;r\right)+\tau_{xy}\;\sin\left(\varepsilon \;\ln\;r\right)\right]\right\}\\ K_{2}=\lim_{r\to 0}\left\{\sqrt{2\pi r}\left[\tau_{xy}\;\cos\left(\varepsilon \;\ln\;r\right)-\sigma_{y}\;\sin\left(\varepsilon \;\ln\;r\right)\right]\right\} \end{array}$$

## 6 Numerical cases

Both logarithmic and Gaussian numerical integrals using 4 integration points can achieve better results through numerical testing. The proposed method is compared with other developed BEM. The results in literatures have been verified by various methods and taken as reference solutions.

### 6.1 Case 1. Results for a finite rectangular plate containing a center crack

**Fig. 4:**
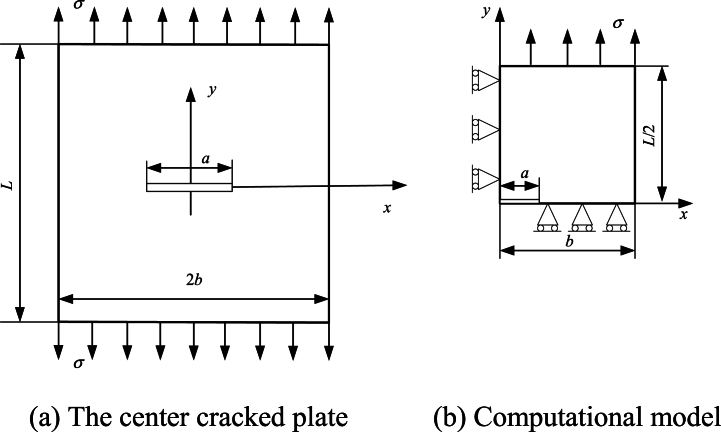
The center cracked plate and its computational model.

A crack problem in mode I is used as the first numerical case. The geometry model of central crack plate is shown in Fig. 4(a), with length 2*b*, width *L* and center crack width 2*a*. The plate is subjected to the plane stress condition of symmetric uniform load σ. According to symmetry, the model can be simplified as shown in Fig. 4(b).

The analytical solution of non-dimensional SIF is given in Ref. [40], as shown in Eq. (37):

(37) $$F\left(a/b\right)=K_{1}/\sigma \sqrt{\pi a}$$

The F(a/b) is obtained by Laurent series solution is derived by Isida [40] and the value of F(a/b) is given as reference solution.

Then the model in Fig. 4(b) is discretized by 9 BSWI_43_ elements (90 nodes), 45 quadratic BEM elements (90 nodes) and 30 cubic BEM elements (90 nodes), respectively, where quadratic and cubic BEM are denoted as BEM2 and BEM3, respectively. The relative errors of BEM2, BEM3 and BSWI_43_ element relative to the reference solution are shown in Table 1. The maximum relative errors of BSWI_43_, BEM2 and BEM3 are 0.9093 %, 5.772 % and 10.666 %, respectively, indicating that the BSWI_43_ element has higher accuracy than the quadratic and cubic BEM under the same number of nodes.

**Table 1:** Results for normalized SIFs *F* for different *a/b* (*L/2b* = 1).

*a/b*	Isida [40]	BEM2		BEM3		BSWI_43_	
	*F*(*a/b*)	*F*(*a/b*)	*ε* (%)	*F*(*a/b*)	*ε* (%)	*F*(*a/b*)	*ε* (%)
0.1	1.006	0.948	5.772	0.968	3.792	0.997	0.909
0.2	1.025	1.004	1.973	1.016	0.873	1.031	0.602
0.3	1.056	1.057	0.079	1.078	2.120	1.057	0.104
0.4	1.109	1.078	2.816	1.145	3.167	1.105	0.392
0.5	1.187	1.201	1.203	1.274	7.382	1.187	0.054
0.6	1.303	1.352	3.713	1.435	10.123	1.297	0.507
0.7	1.488	1.549	4.060	1.647	10.666	1.494	0.356
0.8	1.816	1.848	1.761	1.971	8.555	1.825	0.502
0.9	2.578	2.465	4.360	2.656	3.022	2.561	0.665

### 6.2 Case 2. Results for a finite rectangular plate containing bilateral crack

**Fig. 5:**
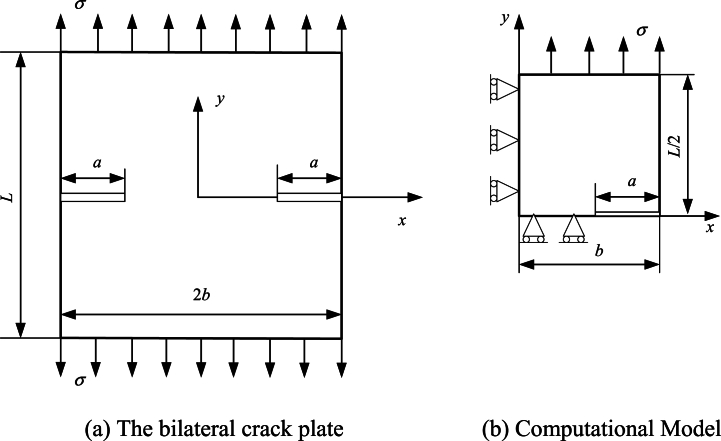
The bilateral crack plate and its computational model.

The bilateral crack plate and corresponding calculation model are shown in Fig. 5.

(38) $$F\left(x\right)=\frac{1.122-0.561x-0.205x^{2}+0.471x^{3}-0.190x^{4}}{\sqrt{1-x}}$$

where x=a/b. The empirical formula (Eq. (38)) for F(x) is provided in Ref. [40], which is called as method 1. Then, 9 BSWI_43_ elements (90 nodes), 45 BEM2 elements (90 nodes) and 30 BEM3 elements (90 nodes) are used to solve this problem, respectively. The relative errors of BEM2, BEM3 and BSWI_43_ element with respect to method 1 are calculated in Table 2.

**Table 2:** Results for normalized SIFs *F* for different *a/b* (*L/2b* = 1).

*a/b*	Method 1	BEM2		BEM3		BSWI_43_	
	*F*(*a/b*)	*F*(*a/b*)	*ε* (%)	*F*(*a/b*)	*ε* (%)	*F*(*a/b*)	*ε* (%)
0.1	1.122	1.031	8.102	0.959	14.539	1.110	1.038
0.2	1.124	1.002	10.867	1.043	7.156	1.110	1.240
0.3	1.131	1.009	10.796	1.090	3.653	1.118	1.202
0.4	1.149	1.090	5.131	1.165	1.344	1.138	1.107
0.5	1.184	1.160	2.028	1.230	3.840	1.190	0.499
0.6	1.247	1.226	1.659	1.292	3.599	1.236	0.925
0.7	1.360	1.309	3.726	1.373	0.997	1.362	0.133
0.8	1.577	1.485	5.863	1.510	4.255	1.561	1.054
0.9	2.118	1.803	14.864	1.856	12.355	2.097	0.983

As shown in Table 2, the maximum relative errors of BSWI_43_, BEM2 and BEM3 are 1.2396 %, 14.864 % and 14.539 %, respectively. Obviously, the error of WBEM with BSWI_43_ is significantly lower than that of the BEM using quadratic and cubic elements.

### 6.3 Case 3. Results for a finite bimaterial rectangular plate containing a center interface crack

**Fig. 6:**
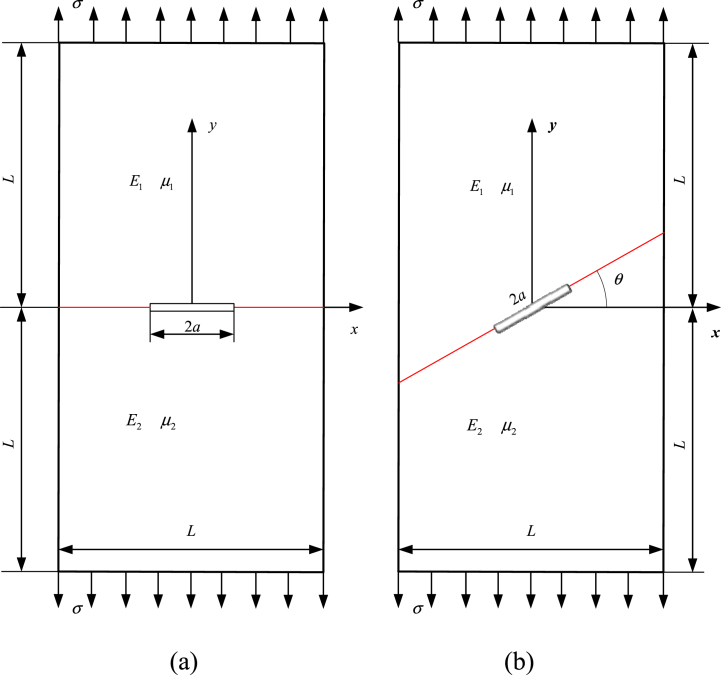
A finite bimaterial plate with (a) center interface crack and (b) slant center interface crack.

A finite bimaterial rectangular plate containing a center interface crack is analyzed in Fig. 6(a), with subjecting uniform stress. The analytical solution of normalized SIF $F_{i}$ is given as

(39) $$F_{i}=K_{i}/\left(\sigma \sqrt{\pi a}\right),\left(i=1,2\right)$$

The $F_{i}$ are evaluated with changing *E1/E2* and *2a/L*, where plane stress condition and $\mu_{1}=\mu_{2}=0.3$ are set. The rectangular plate is divided into two subdomains (seen in Fig. 6(a)), each of which discretized into 4 BSWI_43_ elements. The results of BEM using Hetenyi's fundamental solution is provided in Ref. [17], with 98 quadratic elements for discretization of each subdomain. Table 3 lists the obtained results for the normalized SIFs with changing *E*_1_*/E*_2_ and *2a/L*. The relative error between the reference results and WBEM are computed in Table 3, which indicates WBEM can achieve high-precision results with fewer elements than the quadratic BEM elements with changing *E*_1_*/E*_2_ and *2a/L*.

**Table 3:** Results for normalized SIFs *F*_*i*_ with changing *E*_1_*/E*_2_ and *2a/L*.

*E*_1_*/E*_2_	2*a/L*	Yuuki [17]		WBEM			
		*F*_1_	*F*_2_	F_1_	*ε* (%)	*F*_2_	*ε* (%)
3	0.1	0.989	−0.108	0.991	−0.202	−0.108	0.000
	0.2	1.011	−0.106	1.011	0.000	−0.107	−0.943
	0.3	1.045	−0.107	1.045	0.000	−0.106	0.935
	0.4	1.096	−0.110	1.097	−0.091	−0.111	−0.909
	0.5	1.170	−0.116	1.172	−0.171	−0.114	1.724
	0.6	1.283	−0.127	1.285	−0.156	−0.126	0.787
	0.7	1.459	−0.149	1.495	−2.467	−0.148	0.671
	0.8	1.773	−0.193	1.773	0.000	−0.192	0.518
4	0.1	0.983	−0.129	0.984	−0.102	−0.127	1.550
	0.2	1.005	−0.127	1.004	0.100	−0.128	−0.787
	0.3	1.038	0.127	1.037	0.096	0.128	−0.787
	0.4	1.088	−0.131	1.091	−0.276	−0.132	−0.763
	0.5	1.162	−0.137	1.164	−0.172	−0.135	1.460
	0.6	1.272	−0.151	1.271	0.079	−0.152	−0.662
	0.7	1.445	−0.176	1.447	−0.138	−0.177	−0.568
	0.8	1.751	−0.229	1.753	−0.114	−0.230	−0.437
10	0.1	0.963	−0.173	0.964	−0.104	−0.172	0.578
	0.2	0.985	−0.170	0.986	−0.102	−0.171	−0.588
	0.3	1.018	−0.171	1.017	0.098	−0.172	−0.585
	0.4	1.065	−0.174	1.065	0.000	−0.173	0.575
	0.5	1.134	−0.183	1.135	−0.088	−0.185	−1.093
	0.6	1.238	−0.199	1.237	0.081	−0.200	−0.503
	0.7	1.400	−0.230	1.401	−0.071	−0.228	0.870
	0.8	1.684	−0.295	1.685	−0.059	−0.297	−0.678
100	0.1	0.940	−0.205	0.942	−0.213	−0.206	−0.488
	0.2	0.962	−0.201	0.963	−0.104	−0.202	−0.498
	0.3	0.994	−0.201	0.995	−0.101	−0.202	−0.498
	0.4	1.038	−0.203	1.037	0.096	−0.203	0.000
	0.5	1.104	−0.211	1.106	−0.181	−0.212	−0.474
	0.6	1.201	−0.228	1.202	−0.083	−0.227	0.439
	0.7	1.349	−0.260	1.350	−0.074	−0.261	−0.385
	0.8	1.610	−0.328	1.612	−0.124	−0.329	−0.305

### 6.4 Case 4. Results for a rectangular bimaterial plate with a slant center interface crack

The slant center interface cracks of a finite bimaterial plate shown in Fig. 6(b), where *θ* is slant angle of the crack-faces. The solution domain is divided into two subdomains as shown in Fig. 6(b), where each subdomain is discretized by 4 BSWI_43_ elements. If *2a/L* = 0.5 and $\mu_{1}=\mu_{2}=0.3$, *F*_*i*_ by Eq. (39) are numerically determined as the angle *θ* changes when the elastic modulus ratios *E*_1_/*E*_2_ is 10, 100 and 1000, respectively.

The relative error of WBEM and reference results are given in Table 4, where the reference results are given in Ref. [19]. The absolute value of the highest error is 0.508 %, showing that the present method gives results under coarse mesh matched well with those reported by Gu et al. [19]. Once again, we can see the strong potential of the WBEM for calculating near-tip oscillatory fields.

**Table 4:** The relative error for $F_{i}$ as *θ* and *E*_1_/*E*_2_ changing.

Left/right tip of crack	$\frac{E_{1}}{E_{2}}$	*θ*	*F*_1_			*F*_2_		
			Gu [19]	WBEM	*ε* (%)	Gu [19]	WBEM	*ε* (%)
Left tip of crack	10	15°	1.0096	1.0098	−0.020	−0.4440	−0.4442	−0.045
		30°	0.7843	0.7848	−0.064	−0.6246	−0.6247	−0.016
		45°	0.5217	0.5219	−0.038	−0.6730	−0.6732	−0.030
		60°	0.2772	0.2768	0.144	−0.5810	−0.5818	−0.138
	100	15°	0.9680	0.9683	−0.031	−0.4768	−0.4772	−0.084
		30°	0.7543	0.7546	−0.040	−0.6540	−0.6545	−0.076
		45°	0.5389	0.5390	−0.019	−0.6957	−0.6954	0.043
		60°	0.3498	0.3501	−0.086	−0.5879	−0.5882	−0.051
	1000	15°	0.9597	0.9601	−0.042	−0.4811	−0.4814	−0.062
		30°	0.7493	0.7497	−0.053	−0.6583	−0.6586	−0.046
		45°	0.5454	0.5458	−0.073	−0.6965	−0.6967	−0.029
		60°	0.3691	0.3696	−0.135	−0.5749	−0.5752	−0.052
Right tip of crack	10	15°	1.1269	1.1271	−0.018	0.0852	0.0854	−0.235
		30°	0.9924	0.9925	−0.010	0.2949	0.2951	−0.068
		45°	0.7656	0.7655	0.013	0.4061	0.4064	−0.074
		60°	0.4919	0.4920	−0.020	0.4059	0.4062	−0.074
	100	15°	1.1155	1.1157	−0.018	0.0590	0.0593	−0.508
		30°	1.0048	1.0046	0.020	0.2619	0.2617	0.076
		45°	0.8029	0.8027	0.025	0.3556	0.3559	−0.084
		60°	0.5284	0.5286	−0.038	0.3290	0.3292	−0.061
	1000	15°	1.1164	1.1165	−0.009	0.0574	0.0576	−0.348
		30°	1.0075	1.0076	−0.010	0.2596	0.2598	−0.077
		45°	0.8065	0.8067	−0.025	0.3471	0.3476	−0.144
		60°	0.5221	0.5225	−0.077	0.3055	0.3057	−0.065

### 6.5 Case 5. Results for a bimaterial rectangular plate with an edge interface crack

**Fig. 7:**
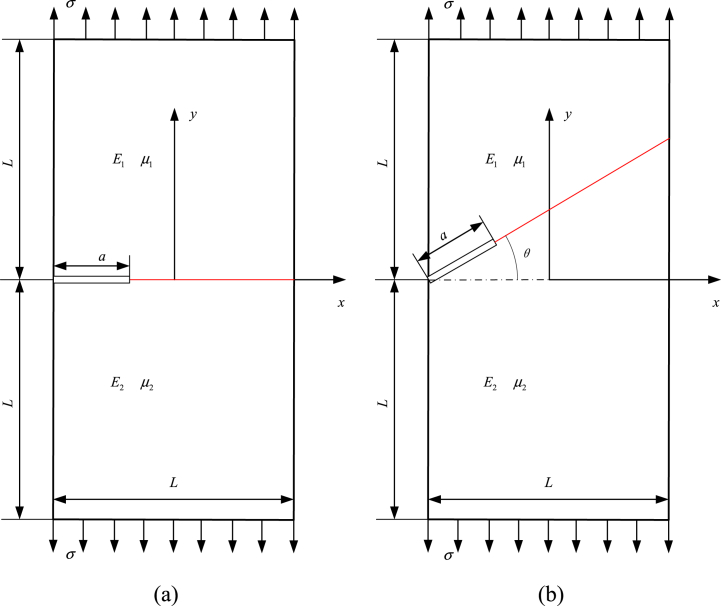
A rectangular bimaterial plate with (a) edge interface crack and (b) slant edge interface crack.

To verify the generality of the method, an edge interface crack between rectangular different material is shown in Fig. 7(a). The plane stress and Possion's ratio $\mu_{1}=\mu_{2}=0.3$ are assumed. Then, 4 BSWI_43_ elements are used for each subdomain. The reference results are obtained in Ref. [42] based on M_1_-integral. Table 5 lists the results for the normalized SIFs obtained by changing *E*_1_*/E*_2_ and *a/L*. The results are given by Matsumto et al. [18] applying the BEM sensitivity analysis and the interaction energy release rates. The relative errors between WBEM and the reference results [42] are calculated in Table 5. The absolute value of highest error is 1.600 %, which indicates that the present results have good agreements with the reference results. The method in this paper still maintains a high accuracy for SIFs under coarse mesh.

**Table 5:** Results for $F_{i}=K_{i}/\left(\sigma \sqrt{\pi a}\right)$, (*i* = 1,2) with changing *E*_1_*/E*_2_ and *a/L*.

*E*_1_/*E*_2_	*a*/*L*	Miyazaki [42]		Matsumto [18]		WBEM			
		*F*_1_	*F*_2_	*F*_1_	*F*_2_	*F*_1_	*ε* (%)	*F*_2_	*ε* (%)
4	0.1	1.209	−0.239	1.199	−0.237	1.211	−0.165	−0.241	−0.837
	0.2	1.368	−0.250	1.368	−0.251	1.369	−0.073	−0.254	−1.600
	0.3	1.654	−0.288	1.655	−0.288	1.656	−0.121	−0.289	−0.347
	0.4	2.101	−0.359	2.102	−0.358	2.107	−0.286	−0.362	−0.836
	0.5	2.807	−0.483	2.806	−0.483	2.810	−0.107	−0.488	−1.035
	0.6	4.006	−0.716	4.001	−0.714	4.006	0.000	−0.717	−0.140
	0.7	6.304	−1.208	6.298	−1.204	6.304	0.000	−1.204	0.331
	0.8	11.820	−2.538	11.780	−2.515	11.821	−0.008	−2.537	0.039
10	0.1	1.229	−0.340	1.222	−0.336	1.228	0.081	−0.341	−0.294
	0.2	1.369	−0.349	1.366	−0.348	1.364	0.365	−0.351	−0.573
	0.3	1.648	−0.399	1.648	−0.394	1.649	−0.061	−0.401	−0.501
	0.4	2.090	−0.494	2.090	−0.491	2.092	−0.096	−0.495	−0.202
	0.5	2.789	−0.663	2.789	−0.661	2.787	0.072	−0.668	−0.754
	0.6	3.974	−0.978	3.968	−0.973	3.973	0.025	−0.977	0.102
	0.7	6.241	−1.648	6.227	−1.634	6.245	−0.064	−1.646	0.121
	0.8	11.660	−3.456	11.590	−3.414	11.661	−0.009	−3.457	−0.029
100	0.1	1.251	−0.424	1.251	−0.424	1.252	−0.080	−0.425	−0.236
	0.2	1.370	−0.428	1.376	−0.429	1.371	−0.073	−0.429	−0.234
	0.3	1.642	−0.485	1.647	−0.470	1.643	−0.061	−0.486	−0.206
	0.4	2.078	−0.597	2.083	−0.569	2.077	0.048	−0.596	0.168
	0.5	2.770	−0.797	2.772	−0.793	2.771	−0.036	−0.796	0.125
	0.6	3.940	−1.172	3.906	−1.171	3.941	−0.025	−1.173	−0.085
	0.7	6.177	−1.969	6.157	−1.957	6.178	−0.016	−1.971	−0.102
	0.8	11.500	−4.124	11.430	−4.075	11.504	−0.035	−4.115	0.218

### 6.6 Case 6. Results for a bimaterial rectangular plate containing a slant edge interface crack

A slant edge crack between dissimilar media is shown in Fig. 7(b). The normalized SIFs are computed using WBEM and Eq. (35). The results for the normalized SIFs are evaluated for several values of *E*_1_*/E*_2_ and *a/L*. The boundary of each subdomain is discretized by 16 BSWI_43_ elements.

Table 6 list results in comparison with the results by the displacement extrapolation method with 100 BEM elements [18]. The highest error by WBEM is 1.087 %, which verifies that WBEM can still yield reliable SIFs solutions in a slant edge interface crack problem under coarse grid.

**Table 6:** Results for $F_{i}=K_{i}/\left(\sigma \sqrt{\pi a}\right)$, (*i* = 1,2) with changing *E*_1_*/E*_2_ and *a/L*.

*E*_1_*/E*_2_	*a/L*	Extrapolation [18]		WBEM			
		*F*_1_	*F*_2_	*F*_1_	*ε* (%)	*F*_2_	*ε* (%)
10	0.2	1.153	0.238	1.154	−0.087	0.236	0.422
	0.5	2.216	0.185	2.216	−0.045	0.186	−1.087
100	0.2	1.163	0.232	1.163	−0.086	0.232	−0.433
	0.5	2.274	0.093	2.272	0.044	0.093	−1.087

## 7 Conclusions

This article constructs a crack-tip element based on wavelet basis functions and combines multi-domain methods to achieve crack analysis. The accuracy and effectiveness of the method were verified through numerical examples. The main characteristics of this method can be summarized as follows.

(1) The constructed crack-tip element can effectively approximate the oscillatory displacement and stress field near the crack tip. High-precision SIFs for interface cracks can be obtained through multi-domain WBEM.
(2) This method is easy to implement and can directly evaluate the SIFs near the interface crack tip.
(3) Even in cases where the boundary mesh is relatively rough, the proposed method can achieve higher computational accuracy than traditional BEM.
The proposed approach was able to analyze crack of homogeneous material and interface cracks of composite bimaterials. Although the current method is developed for 2D crack problems, extending this method to 3D crack problems is well-understood. In addition, the method can be further used to simulate fatigue crack propagation and predict fatigue life.

## CRediT authorship contribution statement

**Jiaxing Chen:** Writing – original draft, Methodology, Investigation. **Dongjie Yuan:** Visualization, Validation, Software. **Ronggang Yang:** Supervision, Funding acquisition. **Jiawei Xiang:** Supervision, Project administration, Funding acquisition, Conceptualization, Writing – review & editing.

## Declaration of competing interest

The authors declare that they have no known competing financial interests or personal relationships that could have appeared to influence the work reported in this paper.
